# Testing for Hepatitis C During Pregnancy Among Persons With Medicaid and Commercial Insurance: Cohort Study

**DOI:** 10.2196/40783

**Published:** 2023-09-27

**Authors:** Mohammed A Khan, William W Thompson, Ademola Osinubi, William A Meyer 3rd, Harvey W Kaufman, Paige A Armstrong, Monique A Foster, Noele P Nelson, Carolyn Wester

**Affiliations:** 1 Division of Viral Hepaitits Centers for Disease Control and Prevention Atlanta, GA United States; 2 Quest Diagnostics Secaucus, NJ United States

**Keywords:** hepatitis C, testing, pregnancy, pregnant, trend, insurance, insured, HCV, hepatitis, Medicaid, health coverage, maternal, fetus, birth, natal, maternity, liver, communicable disease, viral infection

## Abstract

**Background:**

The reported incidence of acute hepatitis C virus (HCV) infection is increasing among persons of childbearing age in the United States. Infants born to pregnant persons with HCV infection are at risk for perinatal HCV acquisition. In 2020, the United States Preventive Services Task Force and Centers for Disease Control and Prevention recommended that all pregnant persons be screened during each pregnancy for hepatitis C. However, there are limited data on trends in hepatitis C testing during pregnancy.

**Objective:**

We estimated hepatitis C testing rates in a large cohort of patients with Medicaid and commercial insurance who gave birth during 2015-2019 and described demographic and risk-based factors associated with testing.

**Methods:**

Medicaid and commercial insurance claims for patients aged 15-44 years and who gave birth between 2015 and 2019 were included. Birth claims were identified using procedure and diagnosis codes for vaginal or cesarean delivery. Hepatitis C testing was defined as an insurance claim during the 42 weeks before delivery. Testing rates were calculated among patients who delivered and among the subset of patients who were continuously enrolled for 42 weeks before delivery. We also compared the timing of testing relative to delivery among patients with commercial or Medicaid insurance. Multivariable logistic regression was used to identify factors associated with testing.

**Results:**

Among 1,142,770 Medicaid patients and 1,207,132 commercially insured patients, 175,223 (15.3%) and 221,436 (18.3%) were tested for hepatitis C during pregnancy, respectively. Testing rates were 89,730 (21.8%) and 187,819 (21.9%) among continuously enrolled Medicaid and commercially insured patients, respectively. Rates increased from 2015 through 2019 among Medicaid (from 20,758/108,332, 19.2% to 13,971/52,330, 26.8%) and commercially insured patients (from 38,308/211,555, 18.1% to 39,152/139,972, 28%), respectively. Among Medicaid patients, non-Hispanic Black (odds ratio 0.73, 95% CI 0.71-0.74) and Hispanic (odds ratio 0.53, 95% CI 0.51-0.56) race or ethnicity were associated with lower odds of testing. Opioid use disorder, HIV infection, and high-risk pregnancy were associated with higher odds of testing in both Medicaid and commercially insured patients.

**Conclusions:**

Hepatitis C testing during pregnancy increased from 2015 through 2019 among patients with Medicaid and commercial insurance, although tremendous opportunity for improvement remains. Interventions to increase testing among pregnant persons are needed.

## Introduction

Chronic hepatitis C virus (HCV) infection is a leading cause of liver disease and liver-related mortality in the United States. Approximately 2.4 million persons in the United States were living with chronic hepatitis C during 2013-2016 [1,2]. These patients are at increased risk of progression to liver fibrosis, cirrhosis, liver failure, hepatocellular carcinoma, and death [3]. HCV can be transmitted during pregnancy or delivery—an estimated 5.8% of infants born to women with HCV infection become infected [4]. Perinatal infection can result in liver disease during adulthood and sequelae such as cirrhosis and hepatocellular carcinoma [5]. The incidence of acute HCV infection in the United States increased annually from 2009 to 2019, with rates highest among adults 20-29 and 30-39 years of age, including women of childbearing age [6,7]. These increases are concurrent with increases in the nation’s opioid crisis [8]. Recent studies have documented an increasing prevalence of HCV infection among pregnant persons [9]. In 2018, 0.5% of all live births nationally were among mothers with hepatitis C [10]. The rate of maternal HCV infection as recorded on US birth certificates increased by 89% between 2009 and 2014, and rates were highest in Appalachia [11]. Hepatitis C antibody testing among pregnant persons from a large national commercial laboratory increased from 5.7% to 13.4% and test positivity increased from 2.6% to 3.6% from 2011 to 2016, respectively [12].

While chronic HCV infection can be cured with direct-acting antiviral therapy, direct-acting antiviral therapies have not been approved for use during pregnancy by the US Food and Drug Administration [13]. However, identifying hepatitis C during pregnancy offers several advantages, including referral for care and treatment for the pregnant person and referral for testing for the exposed infant and can inform clinical decision-making during prenatal and intrapartum care [14]. Since 2012 and 2013, the Centers for Disease Control and Prevention (CDC) and the United States Preventive Services Task Force (USPSTF), respectively, have recommended 1-time hepatitis C testing for all persons born from 1945 through 1965 and testing adults with risk factors for HCV infection [15]. In 2018, the American Association for the Study of Liver Diseases and the Infectious Diseases Society of America recommended hepatitis C screening during each pregnancy [13]. In 2020, the USPSTF and CDC recommended that all pregnant persons be screened during each pregnancy for hepatitis C [14]. However, there are limited data on trends in hepatitis C testing during pregnancy. We estimated hepatitis C testing rates in a large cohort of patients with Medicaid and commercial insurance who gave birth during 2015-2019 and described demographic and risk-based factors associated with testing.

## Methods

### Overview

Data for persons with commercial, Medicare Supplemental, and Medicaid insurance were obtained from the Merative MarketScan Research Databases. These data include demographics, insurance enrollment information, inpatient and outpatient, and prescription drug claims. The commercial and Medicare Supplemental databases include US persons with employer-sponsored health insurance coverage and their dependents. The MarketScan Multi-State Medicaid database includes claims for patients enrolled in Medicaid fee-for-service and managed care plans in multiple states; the number and names of states are proprietary and were not disclosed. Data from January 1, 2014, to December 31, 2019, were used to allow for a 42-week baseline enrollment period prior to delivery between 2015 and 2019.

### Cohort Selection

We constructed a cohort of female patients aged 15-44 years who gave birth from January 1, 2015, to December 31, 2019. Birth claims were identified using Current Procedural Terminology (CPT) and Diagnosis-Related Group codes specific to vaginal or cesarean delivery (Table S1 in Multimedia Appendix 1). Only the earliest claim for delivery during the study period was retained; the service date of this claim was the patient’s index delivery date. As hepatitis C testing during every pregnancy was not recommended during the study period, patients whose HCV infection was identified in the first pregnancy may not have been tested in subsequent pregnancies. Thus, persons with multiple births during the study period were counted once at their earliest delivery.

### Measures

Claims for hepatitis C testing were identified using CPT codes for any anti-HCV antibody, RNA, and genotyping tests. The anti-HCV antibody test indicates past or current HCV infection, while HCV RNA or genotyping test identifies or characterizes current HCV infection. Screening with the HCV antibody test is recommended [14]. Hepatitis C testing during pregnancy was defined as the earliest hepatitis C testing claim in the 42 weeks prior to the index delivery date. Demographic characteristics included age, race or ethnicity (Medicaid only), and region of residence (commercial only). Clinical characteristics (alcohol use disorder, opioid use disorder, severe mental illness, obesity, HIV infection, gestational diabetes, preeclampsia, preterm labor, and singleton or multiple pregnancy) were identified using the International Classification of Diseases, Ninth/Tenth Revision, Clinical Modification (ICD-9/10-CM) codes associated with a claim in the 42 weeks prior to the index delivery date. Additional prenatal care characteristics included an obstetric test panel (including complete blood count, hepatitis B surface antigen, rubella antibody, nontreponemal syphilis test, ABO blood group and Rh typing, and red blood cell antibody screen) claim and influenza and tetanus-diphtheria-acellular pertussis (Tdap) vaccination during the 42 weeks before the index delivery date and were defined using CPT codes (Table S1 in Multimedia Appendix 1).

### Statistical Analysis

Due to incomplete follow-up during the 42 weeks before the index delivery date, we estimated the rate of hepatitis C testing during pregnancy using two methods: (1) percent tested among all patients who gave birth during the study period regardless of baseline enrollment criteria and (2) percent tested among patients continuously enrolled for 42-week period prior to the index delivery date. In sensitivity analyses, we explored the impact of (1) changing the continuous enrollment criteria (12, 24, and 36 weeks, and median time to hepatitis C test), (2) restricting the sample to patients who had an obstetric panel test claim in the 42 weeks before the index delivery date, and (3) restricting the sample to patients who had any inpatient or outpatient service claim in the 42 weeks prior to the index delivery date on the estimated testing rate. The cumulative incidence of testing among patients continuously enrolled for at least 7 days before the index delivery date was also estimated using the Kaplan-Meier method. The Kaplan-Meier method can be used to estimate testing incidence in the presence of censoring (ie, incomplete follow-up). We included a 7-day enrollment minimum to exclude outliers and ensure some demographic and clinical data would be available.

We estimated the testing rate by year of delivery for each cohort and tested for statistically significant trends using the Cochran-Armitage test or for cumulative incidence estimates, and the Gray test for equality of cumulative incidence functions. Additionally, we examined the distribution of time between hepatitis C testing and index delivery date by year of delivery and insurance type. Demographic and clinical characteristics for cohorts constructed using both methods (no enrollment constraint and continuously enrolled for 42 weeks) were described by insurance type and hepatitis C testing status. Multivariable adjusted associations between demographic and clinical characteristics were estimated using logistic regression with the outcome defined as a claim for hepatitis C testing during the 42-week period prior to the index delivery date. We included all variables in multivariable models because our analysis was primarily exploratory and causal relationships between these variables and hepatitis C testing are poorly understood. Odds ratios and 95% CIs are provided. Adjusted marginal probabilities were also estimated. In a sensitivity analysis, logistic regression was performed for patients who had an obstetric panel test insurance claim. Analyses were performed in SAS (version 9.4; SAS Institute).

### Ethical Considerations

This analysis of deidentified data did not require institutional review board approval.

## Results

### Overview

We identified 1,152,283 patients with Medicaid and 1,217,686 patients with commercial insurance who had delivery claims between 2015 and 2019. Among these persons, 1,142,770 (99.2%) Medicaid patients and 1,207,132 (99.1%) commercially insured patients had valid demographic and enrollment information. When restricting the analyses to patients who were continuously insurance enrolled for 42 weeks prior to the index delivery date, 411,795 (36%) Medicaid patients and 857,618 (71%) commercially insured patients remained. Among all Medicaid patients, 175,223 (15.3%) were tested for hepatitis C in the 42 weeks prior to the index delivery date; among the continuously enrolled subset, 89,730 (21.8%) were tested. Among all commercially insured patients, 221,436 (18.3%) were tested for hepatitis C before the index delivery date, and among the continuously enrolled subset, 187,819 (21.9%) were tested.

**Figure 1:**
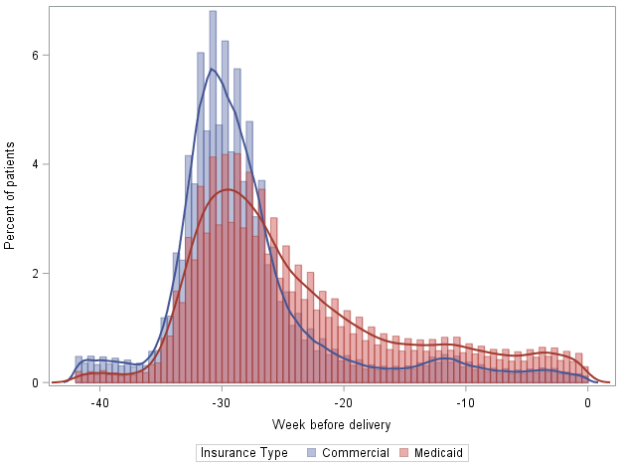
Distribution of weeks between hepatitis C test and delivery date, MarketScan Medicaid and Commercial.

A majority of tested Medicaid (162,868/175,223, 93%) and commercially insured (217,969/221,436, 98.5%) patients were tested for HCV antibody only. The median time between hepatitis C testing and index delivery date for Medicaid patients was 186 (IQR 137-211) days (Figure 1). For commercial insurance patients, the median time between hepatitis C testing and index delivery date was 206 (IQR 184-222) days, suggesting they were tested on average 20 days earlier than Medicaid patients.

### Trends in Hepatitis C Testing

**Figure 2:**
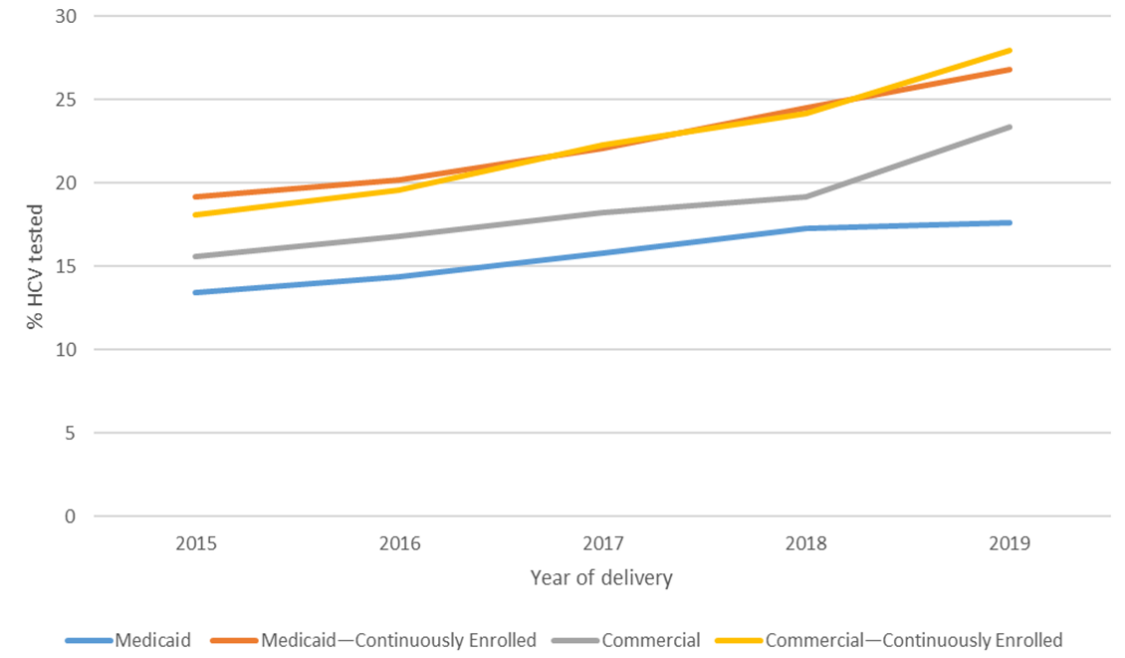
Hepatitis C testing by year of delivery and insurance type. HCV: hepatitis C virus.

The hepatitis C testing rate increased from 2015 to 2019 for both Medicaid and commercially insured patients (Figure 2). Among Medicaid patients, testing rates increased from 13.4% (40,351/301,849) in 2015 to 17.6% (31,123/177,004) in 2019, and among continuously enrolled persons, testing rates increased from 19.2% (20,758/108,332) in 2015 to 26.8% (13,971/52,230) in 2019. Among commercially insured patients, the testing rates increased from 15.6% (43,941/281,007) in 2015 to 23.4% (45,930/296,344) in 2019, and among continuously enrolled persons, the testing rates increased from 18.1% (38,308/211,555) in 2015 to 28% (39,152/139,972) in 2019. Trends in testing were similar in the sensitivity analysis using different continuous enrollment thresholds and testing rate estimation methods: testing rates increased between 2015 and 2019 across all methods. Among patients with Medicaid insurance, the average annual change in testing rate varied from 0.8% for the cohort with no enrollment constraints to 1.7% for the cohorts with an obstetric panel claim. Among patients with commercial insurance, the average annual change in testing rate varied from 1.6% for the cohort with no enrollment constraints to 2% for the Kaplan-Meier estimate (Table S3 in Multimedia Appendix 1).

### Characteristics of Continuously Enrolled Cohorts

Demographic characteristics of patients by insurance type and hepatitis C testing status for the continuously enrolled cohorts are included in Table 1. Patients with Medicaid insurance in the continuously enrolled cohort were predominantly aged 19-29 years (265,421/411,795, 64.5%) followed by those aged 30-39 years (107,665/411,795, 26.2%). The distribution of race and ethnicity was as follows: 48% (196,441/411,795) of Medicaid patients were non-Hispanic White, followed by non-Hispanic Black (152,700/411,795, 37.1%), other race and ethnicity (44,891/411,795, 10.9%), Hispanic, all races (15,347/411,795, 3.7%), and unknown race or ethnicity (2416/411,795, 0.6%). High-risk pregnancy (202,584/411,795, 49.2%), obesity (78,781/411,795, 19.1%), preterm labor (63,219/411,795, 15.4%), severe mental illness (52,133/411,795, 12.7%), and gestational diabetes (35,249/411,795, 8.6%) were the most frequently occurring underlying conditions (Table 2). Forty-three percent (175,695/411,795) of patients received an obstetric panel test; 28.7% (117,969/411,795) and 15.7% (64,433/411,795) received Tdap and influenza vaccinations, respectively.

On average, commercially insured patients in the continuously enrolled cohort were older than the Medicaid patients. Among patients with commercial insurance, those aged 30-39 years (494,564/857,618, 57.7%) represented the largest age group, which is followed by those aged 19-29 years (315,311/857,618, 36.8%). In terms of geographic distribution, patients resided in the south (387,766/857,618, 45.2%), north central (177,013/857,618, 20.6%), west (148,048/857,618, 17.3%), and northeast (142,531/857,618, 16.6%). The distribution of underlying conditions also differed for the commercial sample relative to those with Medicaid: high-risk pregnancy (361,659/857,618, 42.2%), obesity (103,780/857,618, 12.1%), gestational diabetes (90,884/857,618, 10.6%), and preterm labor (73,004/857,618, 8.5%) were the most frequently occurring underlying conditions. Opioid use disorder (2443/857,618, 0.3%) and alcohol use disorder (1656/857,618, 0.2%) were less common than among Medicaid patients (14,069/411,795, 3.4% and 4973/411,795 1.2%, respectively). Claims for an obstetric panel test (470,404/857,618, 54.9%), Tdap vaccination (455,485/857,618, 53.1%), and influenza vaccination (258,427/857,618, 29.1%) were more common among commercially insured patients.

**Table 1:** Demographic and clinical characteristics by hepatitis C virus testing status for patients who were continuously enrolled for 42 weeks, MarketScan commercial and Medicaid, 2015-2019.

			Medicaid				Commercial		
			Total (N=411,795)	Tested (n=89,730)	Not tested (n=322,065)	Total (N=857,618)		Tested (n=187,819)	Not Tested (n=669,799)
**Age group (years), n (%)**									
	15-18		31,636 (7.7)	6578 (7.5)	24,878 (7.7)	6035 (0.7)		1308 (0.7)	4727 (0.7)
	19-29		265,421 (64.5)	57,570 (64.2)	207,851 (65.5)	315,311 (36.8)		67,827 (36.1)	247,484 (37)
	30-39		107,665 (26.2)	23,934 (26.7)	83,731 (26)	494,564 (57.7)		108,716 (57.9)	385,848 (57.6)
	40-44		7073 (1.7)	1468 (1.6)	5605 (1.7)	41,708 (4.9)		9968 (5.3)	31,740 (4.7)
**Race and ethnicity, n (%)**									
		Non-Hispanic Black	152,700 (37.1)	27,828 (31)	124,872 (38.8)	N/A^a^		N/A	N/A
		Hispanic	15,347 (3.7)	2056 (2.3)	13,291 (4.1)	N/A		N/A	N/A
		Non-Hispanic White	196,441 (47.7)	49,309 (55)	147,132 (45.7)	N/A		N/A	N/A
		Other	44,891 (10.9)	9900 (11)	34,991 (10.9)	N/A		N/A	N/A
		Unknown	2416 (0.6)	637 (0.7)	1779 (0.6)	N/A		N/A	N/A
**Region, n (%)**									
		Northeast	N/A	N/A	N/A	142,531 (16.6)		41,447 (22.1)	101,084 (15.1)
		North central	N/A	N/A	N/A	177,013 (20.6)		24,525 (13.1)	152,488 (22.8)
		South	N/A	N/A	N/A	387,766 (45.2)		96,721 (51.5)	291,045 (43.5)
		West	N/A	N/A	N/A	148,048 (17.3)		24,586 (13.1)	123,462 (18.4)
		Unknown	N/A	N/A	N/A	2260 (0.3)		540 (0.3)	1720 (0.3)

^a^N/A: not available.

**Table 2:** Clinical characteristics by hepatitis C virus testing status for patients who were continuously enrolled for 42 weeks, MarketScan Commercial and Medicaid, 2015-2019.

	Medicaid				Commercial		
	Total (N=411,795), n (%)	Tested (n=89,730), n (%)	Not tested (n=322,065), n (%)	Total (N=857,618), n (%)		Tested (n=187,819), n (%)	Not tested (n=669,799), n (%)
Alcohol use disorder	4973 (1.2)	1854 (2.1)	3119 (1)	1656 (0.2)		524 (0.3)	1132 (0.2)
Opioid use disorder	14,069 (3.4)	7767 (8.9)	6302 (2)	2443 (0.3)		1021 (0.5)	1422 (0.2)
Severe mental illness	52,133 (12.7)	15,181 (16.9)	36,952 (11.5)	37,393 (4.4)		9157 (4.9)	28,236 (4.2)
Obesity	78,781 (19.1)	17,736 (19.8)	61,045 (19)	103,780 (12.1)		26,224 (14)	77,556 (11.6)
HIV infection	879 (0.2)	525 (0.6)	354 (0.1)	784 (0.1)		320 (0.2)	464 (0.1)
Preeclampsia	9467 (2.3)	2280 (2.5)	7187 (2.2)	22,019 (2.6)		5528 (2.9)	16,491 (2.5)
High-risk pregnancy	202,584 (49.2)	51,588 (57.5)	150,996 (46.9)	361,659 (42.2)		87,263 (46.5)	274,396 (41)
Gestational diabetes	35,249 (8.6)	7888 (8.8)	27,361 (8.5)	90,884 (10.6)		21,094 (11.2)	69,790 (10.4)
Preterm labor	63,219 (15.4)	14,286 (15.9)	48,933 (15.2)	73,004 (8.5)		16,759 (8.9)	56,245 (8.4)
Multiple gestation	9404 (2.3)	1992 (2.2)	7412 (2.3)	24,791 (2.9)		6106 (3.3)	18,685 (2.8)
Obstetric panel	175,695 (42.7)	41,930 (46.7)	133,765 (41.5)	470,404 (54.9)		109,830 (58.5)	360,574 (53.8)
Tdap^a^ vaccination	117,969 (28.7)	24,452 (27.3)	93,517 (29)	455,485 (53.1)		95,270 (50.7)	360,215 (53.8)
Influenza vaccination	64,433 (15.7)	13,784 (15.4)	50,649 (15.7)	258,427 (30.1)		54,608 (29.1)	203,819 (30.4)

^a^Tdap: tetanus-diphtheria-acellular pertussis.

### Factors Associated With Testing Among Continuously Enrolled Patients

Hepatitis C testing status varied by patient characteristics. Adjusted associations between patient characteristics and hepatitis C testing status are presented in Table 3. Among the continuously enrolled Medicaid patients, patients aged 40-44 years (odds ratio [OR] 0.85, 95% CI 0.80-0.90) were less likely to be tested than younger patients. Relative to non-Hispanic White patients, Hispanic patients (OR 0.53, 95% CI 0.51-0.56), non-Hispanic Black (OR 0.73, 95% CI 0.71-0.74), and other race (OR 0.90, 95% CI 0.88-0.92) were less likely to be tested among Medicaid patients.

Patients with opioid use disorder (OR 3.75, 95% CI 3.62-3.89) or HIV infection (OR 5.40, 95% CI 4.71-6.20) claims were significantly more likely to be tested than those without these conditions. Patients with severe mental illness claims or alcohol use disorder were 1.28 (95% CI 1.25-1.30) and 1.29 (95% CI 1.21-1.37) times more likely to be tested than those without these conditions. High-risk pregnancy (OR 1.46, 95% CI 1.44-1.48) was associated with higher odds of testing, whereas multiple gestations (OR 0.91, 95% CI 0.86-0.96) was associated with lower odds of testing compared to patients without these characteristics.

Among patients with commercial insurance, patients living in the north central (OR 0.40, 95% CI 0.39-0.40), west (OR 0.49, 95% CI 0.48-0.50), and south (OR 0.79, 95% CI 0.78-0.80) were less likely to be tested compared to patients residing in the northeast region. Patients with opioid use disorder (OR 2.33, 95% CI 2.15-2.53), HIV infection (OR 1.96, 95% CI 1.70-2.27), and alcohol use disorder (OR 1.36, 95% CI 1.22-1.51) were more likely to be tested than patients without these conditions. High-risk pregnancy (OR 1.23, 95% CI 1.22-1.25), obesity (OR 1.15, 95% CI 1.13-1.17), and multiple gestations (OR 1.11, 95% CI 1.08-1.14) were associated with higher odds of testing compared to patients without these characteristics. Among the cohort of commercially insured patients with no enrollment constraint, effect estimates were higher relative to those with the constraint but overall consistent in the direction of effects. Adjusted marginal probabilities of hepatitis C testing are shown in Table S6 in Multimedia Appendix 1.

**Table 3:** Multivariable-adjusted associations^a^ between demographic and clinical characteristics and hepatitis C testing during pregnancy.

			Medicaid				Commercial		
			No enrollment constraint, OR^b^ (95% CI)		Continuously enrolled for 42 weeks, OR (95% CI)		No enrollment constraint, OR (95% CI)		Continuously enrolled for 42 weeks, OR (95% CI)
**Age group (years)**									
	15-18	1.33 (1.30-1.37)		1.11 (1.07-1.14)		1.18 (1.11-1.25)		1.06 (1.00-1.13)	
	19-29	1.19 (1.17-1.20)		1.08 (1.06-1.10)		1.06 (1.05-1.07)		1.05 (1.04-1.06)	
	30-39	Ref^c^		Ref		Ref		Ref	
	40-44	0.77 (0.74-0.80)		0.85 (0.80-0.90)		0.97 (0.95-0.99)		0.99 (0.97-1.02)	
**Race or ethnicity**									
	Non-Hispanic Black	0.83 (0.82-0.84)		0.73 (0.71-0.74)		N/A^d^		N/A	
	Hispanic	0.43 (0.41-0.44)		0.53 (0.51-0.56)		N/A		N/A	
	Non-Hispanic White	Ref		Ref		N/A		N/A	
	Other	0.88 (0.87-0.90)		0.90 (0.88-0.92)		N/A		N/A	
	Unknown	0.87 (0.84-0.91)		1.09 (0.99-1.19)		N/A		N/A	
**Region**									
	Northeast	N/A		N/A		Ref		Ref	
	North central	N/A		N/A		0.39 (0.39-0.40)		0.40 (0.39-0.40)	
	South	N/A		N/A		0.75 (0.74-0.76)		0.79 (0.78-0.80)	
	West	N/A		N/A		0.48 (0.47-0.49)		0.49 (0.48-0.50)	
	Unknown	N/A		N/A		0.73 (0.66-0.79)		0.77 (0.70-0.85)	
**Clinical characteristics**									
	Alcohol use disorder	1.44 (1.37-1.52)		1.29 (1.21-1.37)		1.45 (1.31-1.60)		1.36 (1.22-1.51)	
	Opioid use disorder	4.46 (4.34-4.58)		3.75 (3.62-3.89)		2.54 (2.35-2.73)		2.33 (2.15-2.53)	
	Severe mental illness	1.48 (1.45-1.50)		1.28 (1.25-1.30)		1.24 (1.22-1.27)		1.15 (1.12-1.17)	
	Obesity	1.17 (1.16-1.19)		1.03 (1.01-1.05)		1.21 (1.19-1.22)		1.15 (1.13-1.17)	
	HIV infection	5.57 (5.05-6.14)		5.40 (4.71-6.20)		2.27 (2.00-2.59)		1.96 (1.70-2.27)	
	Preeclampsia	1.06 (1.03-1.10)		1.07 (1.01-1.12)		1.10 (1.07-1.14)		1.09 (1.06-1.13)	
	High-risk pregnancy	1.83 (1.81-1.85)		1.46 (1.44-1.48)		1.34 (1.33-1.35)		1.23 (1.22-1.25)	
	Gestational diabetes	1.00 (0.98-1.02)		1.00 (0.97-1.02)		1.04 (1.02-1.06)		1.02 (1.01-1.04)	
	Preterm labor	1.08 (1.07-1.10)		1.02 (0.99-1.04)		1.05 (1.02-1.06)		1.02 (1.00-1.04)	
	Multiple gestations	0.91 (0.88-0.94)		0.91 (0.86-0.96)		1.13 (1.10-1.16)		1.11 (1.08-1.14)	
Obstetric panel			1.94 (1.92-1.96)		1.27 (1.25-1.29)		1.80 (1.78-1.82)		1.17 (1.16-1.19)
Tdap^e^ vaccination			0.99 (0.98-1.00)		0.90 (0.88-0.91)		1.03 (1.02-1.04)		0.96 (0.95-0.97)
Influenza vaccination			1.07 (1.06-1.09)		0.99 (0.97-1.02)		1.10 (1.09-1.12)		0.96 (0.95-0.97)

^a^Models included all listed variables.

^b^OR: odds ratio.

^c^Ref: reference.

^d^N/A: not available.

^e^Tdap: tetanus-diphtheria-acellular pertussis.

### Sensitivity Analyses

In the sensitivity analysis of testing rate by inclusion criteria and estimation method, the estimated testing rates increased as the length of the continuous enrollment period increased. The Kaplan-Meier method yielded higher estimates relative to naïve estimates. In addition, patients with an obstetric panel test had higher HCV testing rates relative to those who did not. Overall, differences in estimates across methods and inclusion criteria were larger for the Medicaid patients relative to the commercially insured patients (Table S2 in Multimedia Appendix 1).

In the sensitivity analyses of adjusted associations for HCV testing that included demographic and clinical characteristics, estimates for the Medicaid sample with an obstetric panel test were similar to those who were continuously enrolled for 42 weeks (Table S5 in Multimedia Appendix 1). Among commercially insured patients, the effect estimate for HIV infection was larger (OR 2.61 vs 1.96) for individuals with an obstetric panel test compared to patients enrolled for 42 weeks, although CIs overlapped, and gestational diabetes and preterm labor were not associated with higher odds of testing.

## Discussion

### Principal Findings

We estimated the rates of testing for hepatitis C during pregnancy in a large national sample of patients with Medicaid and commercial insurance using multiple methods. These estimates could serve as a baseline for understanding and monitoring the impact of CDC’s hepatitis C screening recommendations on testing rates during pregnancy. Our results also suggest that patients who are pregnant are most often tested for hepatitis C early in pregnancy. However, Medicaid patients were more likely to be tested closer to the delivery date than commercially insured patients. This is likely linked to later entry into prenatal care as evidenced by the smaller proportion of Medicaid patients continuously enrolled in the 42 weeks prior to delivery, more frequent changes in insurance enrollment (ie, “churn”), or systematic differences in prenatal care practices among providers serving Medicaid patients. It may also be impacted by ongoing risk behaviors (eg, injection drug use) for HCV infection, which might trigger testing when disclosed to the provider. Testing rates for both Medicaid and commercially insured patients increased annually during 2015-2019. As national recommendations for testing during pregnancy did not change during the study period, these trends might reflect the increasing prevalence of or improved ascertainment of hepatitis C risk factors among pregnant persons and increasing awareness among clinicians.

Multiple factors associated with receipt of hepatitis C testing were identified. Results were largely similar for Medicaid and commercially insured patients. Younger patients were more likely to be tested than patients in the oldest age group. Among Medicaid patients, lower rates of testing were identified among Hispanic, non-Hispanic Black, and other non-White non-Hispanic patients. Lower access to prenatal care and quality of prenatal care among racial and ethnic minorities have been extensively reported in the literature [16]. Among commercially insured patients, those residing in the northeast were most likely to be tested. This might reflect regional differences in risk factor prevalence and providers’ testing practices.

Patients with opioid use disorder and HIV infection were approximately 2 to 5 times more likely to be tested. Risk-based testing guidelines recommend testing for these patients; however, current CDC guidance recommends screening all pregnant persons during every pregnancy. Other observed associations such as those with alcohol use disorder and severe mental illness might also reflect unidentified injection drug use. Associations with an obstetric panel test and maternal vaccinations might be due to better retention in or quality of prenatal care. Preterm labor, high-risk pregnancy, obesity, and preeclampsia were also associated with higher odds of testing; these conditions might lead to more intensive prenatal care and inclusive testing practices. Associations with small effect sizes (eg, preterm labor) might also be due to residual confounding.

A single-center study conducted in an urban medical center in 2016 found that only 7% of pregnant patients were tested for hepatitis C [17]. Our estimates were substantially higher and may reflect differences in inclusion criteria (eg, pregnancies versus deliveries) and study populations. Low testing rates might result from a lack of assessment of hepatitis C risk factors and limited reporting of risk factors by pregnant individuals. Previous studies have reported that 10% to 59% of anti-HCV–positive pregnant persons reported no risk factors and that approximately two-thirds of patients who are pregnant reporting risk factors were not tested for hepatitis C [17,18]. A recent study of commercial laboratory test data found that 18% and 25% of obstetric panels for commercial and Medicaid patients, respectively, also had an HCV antibody test within a year [19]. Estimates of the hepatitis C testing rate were sensitive to inclusion criteria (eg, continuous enrollment thresholds). This was particularly evident for Medicaid patients for whom testing rates ranged from 15.3% to 23.9%. Continuously enrolled Medicaid and commercially insured patients had similar testing rates. Validation studies are required to assess which method is most accurate. However, the estimates of testing rate trends were robust to different methods of defining the study sample and estimating the testing rate.

### Limitations

This study has several limitations. Administrative data for a very large convenience sample of pregnant persons were used, but we could not evaluate whether this population was representative of all persons who gave birth in the United States. While patients with private insurance and Medicaid were included, women with other forms of insurance or women who were uninsured were not included. The study was also limited to patients who gave birth and did not include pregnancies that ended in spontaneous abortion or termination. Incomplete follow-up was common for this sample and may have resulted in underestimation of testing prevalence if women were tested and then lost to follow-up. Insurance change is relatively common among pregnant persons in the United States; approximately 30% change insurance plans between the preconception period and delivery [20]. We were unable to validate the administrative codes used to identify pregnant persons. Some persons might have been misclassified. The prevalence of obstetric panel testing was lower than expected and might be due to misclassification of pregnancy or testing status (ie, due to missing test panel claims).

These data did not include a measure of socioeconomic status other than insurance type and race or ethnicity (Medicaid only), and variation in testing rate by income, rural and urban status, and so on were not examined. Although procedure codes were used to identify hepatitis C testing, the reason for testing (ie, screening or diagnostic testing) could not be ascertained nor whether testing was motivated by risk factor assessment. Finally, ICD-9/10-CM codes were used to define several clinical characteristics, but we could not assess the sensitivity or specificity of these codes. Additionally, patients with known chronic HCV infection were not excluded from the analysis; this could lead to underestimation of screening rates as these patients should not be included in the denominator.

Despite increases during the study period, testing for hepatitis C remained low in this large national sample of pregnant persons with commercial insurance and Medicaid. The USPSTF and CDC 2020 recommendations for universal hepatitis C screening during each pregnancy might lead to higher rates of testing. Education of providers to ensure testing during each pregnancy, and additional screening for high-risk behaviors that may necessitate repeat testing during pregnancy, are crucial to prevent perinatal transmission of HCV infection. Interventions to increase hepatitis C screening in other populations, such as electronic health record alerts or targeted patient outreach, could be adapted to the prenatal care setting and merit further study [21]. Racial and ethnic disparities in screening rates could be addressed through communication campaigns using trusted messengers to reach racial and ethnic minorities and prenatal care providers that serve these populations. Unrestricted timely treatment of hepatitis C among persons who are of childbearing age would also reduce perinatal transmission [22]. Interventions to increase testing are needed to address perinatal hepatitis C and eliminate hepatitis C in the United States.

## Appendix

Multimedia Appendix 1 Hepatitis C testing among pregnant patients with commercial or Medicaid insurance.

## Abbreviations

CDC: Centers for Disease Control and Prevention
CPT: Current Procedural Terminology
HCV: hepatitis C virus
ICD-9/10-CM: International Classification of Diseases, Ninth/Tenth Revision, Clinical Modification
OR: odds ratio
Tdap: tetanus-diphtheria-acellular pertussis
USPSTF: United States Preventive Services Task Force

## Data Availability

MarketScan data are proprietary and cannot be shared without a purchase agreement with Merative, Inc.
