# Supplementary material for: Testing for Hepatitis C During Pregnancy Among Persons With Medicaid and Commercial Insurance: Cohort Study
Source: JMIR Public Health Surveill. 2023 Sep 27;9:e40783. doi: 10.2196/40783 (PMC10568399; doi:10.2196/40783)
Supplement: Multimedia Appendix 1 [file publichealth_v9i1e40783_app1.docx]

**Supplementary Tables and Figures**

| Table S1. CPT and DRG codes for delivery and hepatitis C testing |  |
| --- | --- |
| **Delivery** | |
| **Description** | **CPT Code** |
| Anesthesia for vaginal delivery only | 1960 |
| Anesthesia for cesarean delivery only | 1961 |
| Neuraxial labor analgesia/anesthesia for planned vaginal delivery (this includes any repeat subarachnoid needle placement and drug injection and/or any necessary replacement of an epidural catheter during labor) | 1967 |
| Anesthesia for cesarean delivery following neuraxial labor analgesia/anesthesia (List separately in addition to code for primary procedure performed) | 1968 |
| Vaginal delivery only (with or without episiotomy and/or forceps); | 59409 |
| Vaginal delivery only (with or without episiotomy and/or forceps); including postpartum care | 59410 |
| Delivery of placenta (separate procedure) | 59414 |
| Cesarean delivery only | 59514 |
| Cesarean delivery only; including postpartum care | 59515 |
| Vaginal delivery only, after previous cesarean delivery (with or without episiotomy and/or forceps) | 59612 |
| Vaginal delivery only, after previous cesarean delivery (with or without episiotomy and/or forceps) including postpartum care | 59614 |
| Cesarean delivery only, following attempted vaginal delivery after previous cesarean delivery | 59620 |
| Cesarean delivery only, following attempted vaginal delivery after previous cesarean delivery; including postpartum care | 59622 |
|  | **DRG Code** |
| Cesarean section with complication or comorbidity/major complication or comorbidity | 765 (2015-2018) |
| Cesarean section without complication or comorbidity/major complication or comorbidity | 766 (2015-2018) |
| Vaginal delivery with sterilization and/or D&C | 767 (2015-2018) |
| Vaginal delivery with O.R. Procedure except sterilization and/or D&C | 768 (2015-2019) |
| Vaginal delivery with complicating diagnoses | 774 (2015-2018) |
| Vaginal delivery without complicating diagnoses | 775 (2015-2018) |
| Cesarean section with sterilization with MCC | 783 (2019) |
| Cesarean section with sterilization with CC | 784 (2019) |
| Cesarean section with sterilization without CC/MCC | 785 (2019) |
| Cesarean section without sterilization with MCC | 786 (2019) |
| Cesarean section without sterilization with CC | 787 (2019) |
| Cesarean section without sterilization without CC/MCC | 788 (2019) |
| Vaginal delivery with sterilization/D&C with MCC | 796 (2019) |
| Vaginal delivery with sterilization/D&C with CC | 797 (2019) |
| Vaginal delivery with sterilization/D&C without CC/MCC | 798 (2019) |
| Vaginal delivery without sterilization/D&C with MCC | 805 (2019) |
| Vaginal delivery without sterilization/D&C with CC | 806 (2019) |
| Vaginal delivery without sterilization/D&C without CC/MCC | 807 (2019) |
|  |  |
| **HCV Testing** | |
| **Description** | **CPT Code** |
| Acute hepatitis panel | 80074 |
| Hepatitis C antibody | 86803 |
| HCV RNA | 87520, 87521, 87522 |
| HCV genotype | 87902 |
|  |  |
| **Clinical Characteristics** | |
| **Description** | **CPT or ICD-9-/ICD-10-CM Code** |
| Tdap vaccination | 90715 |
| Influenza vaccination | 90630, 90653, 90654, 90655, 90656, 90657, 90658, 90661, 90662, 90672, 90673, 90674, 90682, 90685, 90686, 90687, 90689, 9076 |
| Obstetric panel | 80055, 80081 |
| Alcohol use disorder | 291.0, 291.81, 303.00, 303.01, 303.02, 303.03, 303.90, 303.91, 303.92, 303.93, 305.00, 305.01, 305.02, 305.03, F10.10, F10.11, F10.120, F10.121, F10.129, F10.12, F10.14, F10.150, F10.151, F10.159, F10.15, F10.180, F10.181, F10.182, F10.188, F10.18, F10.19, F10.1, F10.20, F10.21, F10.220, F10.221, F10.229, F10.22, F10.230, F10.231, F10.232, F10.239, F10.23, F10.24, F10.250, F10.251, F10.259, F10.25, F10.26, F10.27, F10.280, F10.281, F10.282, F10.288, F10.28, F10.29, F10.2, Z71.41, Z71.4 |
| Opioid use disorder | 304.00, 304.01, 304.02, 304.03, 305.50, 305.51, 305.52, 305.53, 965.01, 965.00, 965.02, 965.09, 304.70, 304.71, 304.72, 304.73, F11.10, F11.11, F11.120, F11.121, F11.122, F11.129, F11.12, F11.14, F11.150, F11.151, F11.159, F11.15, F11.181, F11.182, F11.188, F11.18, F11.19, F11.1, F11.20, F11.21, F11.220, F11.221, F11.222, F11.229, F11.22, F11.23, F11.24, F11.250, F11.251, F11.259, F11.25, F11.281, F11.282, F11.288, F11.28, F11.29, F11.2, F11.90, F11.920, F11.921, F11.922, F11.929, F11.92, F11.93, F11.94, F11.950, F11.951, F11.959, F11.95, F11.981, F11.982, F11.988, F11.98, F11.99, F11.9, F11., T40.2X1, T40.2X1A, T40.2X1D, T40.2X1S, T40.2X2, T40.2X2A, T40.2X2D, T40.2X2S, T40.2X3, T40.2X3A, T40.2X3D, T40.2X3S, T40.2X4, T40.2X4A, T40.2X4D, T40.2X4S, T40.1, T40.1X1, T40.1X1A, T40.1X1D, T40.1X1S, T40.1X2, T40.1X2A, T40.1X2D, T40.1X2S, T40.1X4, T40.1X4A, T40.1X4D, T40.1X4S, T40.1X, T40.3X1, T40.3X1A, T40.3X1D, T40.3X1S, T40.3X2, T40.3X2A, T40.3X2D, T40.3X2S, T40.3X4, T40.3X4A, T40.3X4D, T40.3X4S |
| Severe mental illness | 295.x, 296.x, F20.x, F21.x, F22.x, F23.x, F24.x, F25.x, F26.x, F27.x, F28.x, F29.x, F30.x, F31.x, F32.x, F33.x, F34.x, F35.x, F36.x, F37.x, F38.x, F39.x |
| Obesity | E66.0, E66.01, E66.09, E66.2, E66.3, E66.8, O99.21, O99.210, O99.211, O99.212, O99.213, O99.214, O99.215, 278.0, 278.00, 278.01, 278.02 |
| HIV infection | 042.x, 079.53, V08.x, B20.x |
| Pre-eclampsia | O11.x, O14.x, 642.4, 642.5, 642.6, 642.7 |
| High-risk pregnancy | O09.x, V23.x |
| Gestational diabetes | O244, 648.83 |
| Preterm labor | O60.x, 644.0, 644.1 |
| Multiple gestation | O30.x, 651.x |

| Table S2. Hepatitis C testing during pregnancy by method and insurance type | | | | |  | | | | | |  | | | | | |  | | | |  | |  |
| --- | --- | --- | --- | --- | --- | --- | --- | --- | --- | --- | --- | --- | --- | --- | --- | --- | --- | --- | --- | --- | --- | --- | --- |
|  | | | | | **Medicaid** | | | | | | | | | | | | | | | | | |  |
|  | | | | | **Total** | | | | | | **% Retained** | | | | | | **Tested** | | | | **% Tested** | |  |
| No enrollment constraint | | | | | 1,142,770 | | | | | | 100.0% | | | | | | 175,223 | | | | 15.3% | |  |
| Continuously enrolled for 12 weeks before delivery | | | | | 956,692 | | | | | | 83.7% | | | | | | 166,837 | | | | 17.4% | |  |
| Continuously enrolled for 24 weeks before delivery | | | | | 844,720 | | | | | | 73.9% | | | | | | 157,036 | | | | 18.6% | |  |
| Continuously enrolled for median time to test before delivery | | | | | 809,236 | | | | | | 70.8% | | | | | | 152,966 | | | | 18.9% | |  |
| Continuously enrolled for 36 weeks before delivery | | | | | 534,288 | | | | | | 46.8% | | | | | | 111,041 | | | | 20.8% | |  |
| Continuously enrolled for 42 weeks before delivery | | | | | 411,795 | | | | | | 36.0% | | | | | | 89,730 | | | | 21.8% | |  |
| Obstetric panel claim: No enrollment constraint | | | | | 380,236 | | | | | | 33.3% | | | | | | 83,117 | | | | 21.9% | |  |
| Obstetric panel claim: Continuously enrolled for 42 weeks before delivery | | | | | 175,695 | | | | | | 15.4% | | | | | | 41,930 | | | | 23.9% | |  |
| Any claim in the 42 weeks before delivery | | | | | 1,047,721 | | | | | | 91.7% | | | | | | 175,223 | | | | 16.7% | |  |
| Kaplan-Meier estimate | | | | | 1,052,870 | | | | | | 92.1% | | | | | | 167,025 | | | | 20.9%* | |  |
| *Cumulative incidence | | | | |  | | | | | |  | | | | | |  | | | |  | |  |
|  | | | | | | | | | | | | | | | | | |  |  |  |  |  |  |
|  | **Commercial** | | | | | | | | | | | | | | | | |  |  |  |  |  |  |
|  | **Total** | | | **% Retained** | | | | **Tested** | | | | | | **% Tested** | | | |  |  |  |  |  |  |
| No enrollment constraint | 1,207,132 | | | 100.0% | | | | 221,346 | | | | | | 18.3% | | | |  |  |  |  |  |  |
| Continuously enrolled for 12 weeks before delivery | 1,112,633 | | | 92.2% | | | | 218,791 | | | | | | 19.7% | | | |  |  |  |  |  |  |
| Continuously enrolled for 24 weeks before delivery | 1,018,816 | | | 84.4% | | | | 215,042 | | | | | | 21.1% | | | |  |  |  |  |  |  |
| Continuously enrolled for median time to test before delivery | 971,299 | | | 80.5% | | | | 210,669 | | | | | | 21.7% | | | |  |  |  |  |  |  |
| Continuously enrolled for 36 weeks before delivery | 912,504 | | | 75.6% | | | | 199,834 | | | | | | 21.9% | | | |  |  |  |  |  |  |
| Continuously enrolled for 42 weeks before delivery | 857,618 | | | 71.0% | | | | 187,819 | | | | | | 21.9% | | | |  |  |  |  |  |  |
| Obstetric panel claim: No enrollment constraint | 547,611 | | | 45.4% | | | | 127,902 | | | | | | 23.4% | | | |  |  |  |  |  |  |
| Obstetric panel claim: Continuously enrolled for 42 weeks before delivery | 470,404 | | | 39.0% | | | | 109,830 | | | | | | 23.4% | | | |  |  |  |  |  |  |
| Any claim in the 42 weeks before delivery | 1,190,248 | | | 98.6% | | | | 221,346 | | | | | | 18.6% | | | |  |  |  |  |  |  |
| Kaplan-Meier estimate | 948,943 | | | 78.6% | | | | 218,868 | | | | | | 22.3% | | | |  |  |  |  |  |  |
| *Cumulative incidence | | | | | | | |  | | | | | |  | | | |  |  |  |  |  |  |
|  | | | | | | | |  | | | | | |  | | | |  |  |  |  |  |  |
| Table S3. Trends in hepatitis C testing during pregnancy by year of delivery, method, and insurance type | | | | | | | | | | | | | | | |  | | | |  | |  | |
|  | | | | | | | **Medicaid** | | | | | | | | | | | | | | |  | |
|  | | | | | | | **2015** | | | **2016** | | | **2017** | | | **2018** | | | | **2019** | | **p-value*** | |
| No enrollment constraint | | | | | | | 13.4 | | | 14.4 | | | 15.8 | | | 17.3 | | | | 17.6 | | <.001 | |
| Continuously enrolled for 12 weeks before delivery | | | | | | | 15.1 | | | 16.4 | | | 17.9 | | | 20.0 | | | | 20.6 | | <.001 | |
| Continuously enrolled for 24 weeks before delivery | | | | | | | 16.1 | | | 17.3 | | | 18.9 | | | 21.0 | | | | 22.7 | | <.001 | |
| Continuously enrolled for median time to test before delivery | | | | | | | 16.4 | | | 17.5 | | | 19.2 | | | 21.3 | | | | 23.2 | | <.001 | |
| Continuously enrolled for 36 weeks before delivery | | | | | | | 18.2 | | | 19.4 | | | 21.0 | | | 23.3 | | | | 25.4 | | <.001 | |
| Continuously enrolled for 42 weeks before delivery | | | | | | | 19.2 | | | 20.2 | | | 22.1 | | | 24.5 | | | | 26.8 | | <.001 | |
| Obstetric panel claim: No enrollment constraint | | | | | | | 19.6 | | | 19.9 | | | 21.7 | | | 24.4 | | | | 28.1 | | <.001 | |
| Obstetric panel claim: Continuously enrolled for 42 weeks before delivery | | | | | | | 21.7 | | | 22.0 | | | 23.8 | | | 26.7 | | | | 30.2 | | <.001 | |
| Any claim in the 42 weeks before delivery | | | | | | | 14.7 | | | 15.7 | | | 17.1 | | | 18.8 | | | | 19.3 | | <.001 | |
| Kaplan-Meier estimate | | | | | | | 18.0 | | | 19.3 | | | 21.2 | | | 23.7 | | | | 26.0 | | <.001** | |
| *Cochran-Armitage trend test | | | | | | |  | | |  | | |  | | |  | | | |  | |  | |
| **Gray's test for equality of cumulative incidence functions | | | | | | |  | | |  | | |  | | |  | | | |  | |  | |
|  | | | | | | | | | | | | | | | | | | |  |  |  |  |  |
|  | | **Commercial** | | | | | | | | | | | | |  | | | |  |  |  |  |  |
|  | | **2015** | **2016** | | | **2017** | | | **2018** | | | **2019** | | | **p-value*** | | | |  |  |  |  |  |
| No enrollment constraint | | 15.6 | 16.8 | | | 18.2 | | | 19.2 | | | 23.4 | | | <.001 | | | |  |  |  |  |  |
| Continuously enrolled for 12 weeks before delivery | | 16.5 | 17.9 | | | 19.6 | | | 21.1 | | | 25.0 | | | <.001 | | | |  |  |  |  |  |
| Continuously enrolled for 24 weeks before delivery | | 17.5 | 19.0 | | | 21.2 | | | 23.2 | | | 26.9 | | | <.001 | | | |  |  |  |  |  |
| Continuously enrolled for median time to test before delivery | | 17.9 | 19.4 | | | 21.9 | | | 24.1 | | | 27.6 | | | <.001 | | | |  |  |  |  |  |
| Continuously enrolled for 36 weeks before delivery | | 18.1 | 19.6 | | | 22.3 | | | 24.3 | | | 27.9 | | | <.001 | | | |  |  |  |  |  |
| Continuously enrolled for 42 weeks before delivery | | 18.1 | 19.6 | | | 22.3 | | | 24.2 | | | 28.0 | | | <.001 | | | |  |  |  |  |  |
| Obstetric panel claim: No enrollment constraint | | 19.5 | 20.9 | | | 24.0 | | | 26.3 | | | 29.2 | | | <.001 | | | |  |  |  |  |  |
| Obstetric panel claim: Continuously enrolled for 42 weeks before delivery | | 19.5 | 20.9 | | | 24.2 | | | 26.2 | | | 29.3 | | | <.001 | | | |  |  |  |  |  |
| Any claim in the 42 weeks before delivery | | 15.8 | 17.1 | | | 18.5 | | | 19.5 | | | 23.7 | | | <.001 | | | |  |  |  |  |  |
| Kaplan-Meier estimate | | 18.3 | 19.9 | | | 22.5 | | | 24.8 | | | 28.3 | | | <.001** | | | |  |  |  |  |  |

| Table S4. Demographic and clinical characteristics by HCV testing status, MarketScan Commercial and Medicaid, 2015-2019 | | | | | | | |
| --- | --- | --- | --- | --- | --- | --- | --- |
|  | **Medicaid** | | |  | **Commercial** | | |
|  | **No enrollment constraint** | | |  | **No enrollment constraint** | | |
|  | Total (n=1,142,770) | Tested (n=175,223) | Not Tested (n=967,547) |  | Total (n=1,207,132) | Tested (n=221,346) | Not Tested (n=985,786) |
|  | n (%) | n (%) | n (%) |  | n (%) | n (%) | n (%) |
| Age Group |  |  |  |  |  |  |  |
| 15-18 | 51,353 (4.5) | 9,012 (5.1) | 42,341 (4.4) |  | 7,730 (0.6) | 1,499 (0.7) | 6,231 (0.6) |
| 19-29 | 752,507 (65.9) | 117,305 (67.0) | 635,202 (65.7) |  | 463,249 (38.4) | 82,087 (37.1) | 381,162 (38.7) |
| 30-39 | 314,591 (27.5) | 45,873 (26.2) | 268,718 (27.8) |  | 680,408 (56.4) | 126,410 (57.1) | 553,998 (56.2) |
| 40-44 | 24,319 (2.1) | 3,033 (1.7) | 21,286 (2.2) |  | 55,745 (4.6) | 11,350 (5.1) | 44,395 (4.5) |
|  |  |  |  |  |  |  |  |
| Race/Ethnicity |  |  |  |  |  |  |  |
| Black | 353,261 (30.9) | 51,674 (29.5) | 301,947 (31.2) |  | -- | -- | -- |
| Hispanic | 80,864 (7.1) | 5,227 (3.0) | 75,637 (7.8) |  | -- | -- | -- |
| White | 548,962 (48.0) | 95,913 (54.7) | 453,049 (46.8) |  | -- | -- | -- |
| Other | 132,835 (11.6) | 19,006 (10.9) | 113,829 (11.8) |  | -- | -- | -- |
| Unknown | 26,488 (2.3) | 3,403 (1.9) | 23,085 (2.4) |  | -- | -- | -- |
|  |  |  |  |  |  |  |  |
| Region |  |  |  |  |  |  |  |
| Northeast | -- | -- | -- |  | 195,726 (16.2) | 48,153 (21.8) | 147,573 (15.0) |
| North Central | -- | -- | -- |  | 249,074 (20.6) | 28,505 (12.9) | 220,569 (22.4) |
| South | -- | -- | -- |  | 546,572 (45.3) | 114,790 (51.9) | 431,782 (43.8) |
| West | -- | -- | -- |  | 212,429 (17.6) | 29,268 (13.2) | 183,161 (18.6) |
| Unknown | -- | -- | -- |  | 3,331 (0.3) | 630 (0.3) | 2,701 (0.3) |
|  |  |  |  |  |  |  |  |
| Clinical Characteristics |  |  |  |  |  |  |  |
| Alcohol use disorder | 8,244 (0.7) | 2,863 (1.6) | 5,381 (0.6) |  | 1,991 (0.2) | 599 (0.3) | 1,392 (0.1) |
| Opioid use disorder | 25,140 (2.2) | 12,803 (7.3) | 12,337 (1.3) |  | 3,151 (0.3) | 1,219 (0.5) | 1,932 (0.2) |
| Severe mental illness | 86,110 (7.5) | 22,718 (13.0) | 63,392 (6.6) |  | 45,945 (3.8) | 10,432 (4.7) | 35,513 (3.6) |
| Obesity | 168,625 (14.8) | 32,229 (18.4) | 136,396 (14.1) |  | 135,271 (11.2) | 30,569 (13.8) | 104,702 (10.6) |
| HIV infection | 1,763 (0.2) | 949 (0.5) | 814 (0.1) |  | 1,022 (0.1) | 395 (0.2) | 627 (0.1) |
| Preeclampsia | 23,596 (2.1) | 4,441 (2.5) | 19,155 (2.0) |  | 29,958 (2.5) | 6,560 (3.0) | 23,398 (2.4) |
| High-risk pregnancy | 443,144 (38.8) | 94,185 (53.8) | 348,959 (36.1) |  | 474,538 (39.3) | 101,807 (46.0) | 372,731 (37.8) |
| Gestational diabetes | 87,334 (7.6) | 14,857 (8.5) | 72,477 (7.5) |  | 121,967 (10.1) | 24,651 (11.1) | 97,316 (9.9) |
| Preterm labor | 140,016 (12.3) | 25,569 (14.6) | 114,447 (11.8) |  | 98,747 (8.2) | 19,652 (8.9) | 70,095 (8.0) |
| Multiple gestation | 22,318 (2.0) | 3,680 (2.1) | 18,638 (1.9) |  | 32,669 (2.7) | 7,029 (3.2) | 25,640 (2.6) |
|  |  |  |  |  |  |  |  |
| Obstetric panel | 380,236 (33.3) | 83,117 (47.4) | 297,119 (30.7) |  | 547,611 (45.4) | 127,902 (57.8) | 419,709 (42.6) |
| Tdap vaccination | 283,222 (24.8) | 46,405 (26.5) | 236,817 (24.5) |  | 600,377 (49.7) | 110,578 (50.0) | 489,799 (49.7) |
| Influenza vaccination | 141,451 (12.4) | 24,743 (14.1) | 116,708 (12.1) |  | 306,013 (25.4) | 61,264 (27.7) | 244,749 (24.8) |

| Table S5. Sensitivity analysis: Multivariable-adjusted^a^ associations between demographic and clinical characteristics and hepatitis C testing - obstetric panel only | | | |
| --- | --- | --- | --- |
|  | **Medicaid** |  | **Commercial** |
|  | OR (95% CI) |  | OR (95% CI) |
| Age Group |  |  |  |
| 15-18 | 1.07 (1.03, 1.11) |  | 1.13 (1.05, 1.23) |
| 19-29 | 1.08 (1.06, 1.10) |  | 1.03 (1.02, 1.05) |
| 30-39 | Ref. |  | Ref. |
| 40-44 | 0.84 (0.79, 0.89) |  | 1.02 (0.99, 1.05) |
|  |  |  |  |
| Race/Ethnicity |  |  |  |
| Black | 0.75 (0.74, 0.76) |  | -- |
| Hispanic | 0.67 (0.64, 0.70) |  | -- |
| White | Ref. |  | -- |
| Other | 0.95 (0.92, 0.97) |  | -- |
| Unknown | 1.36 (1.27, 1.45) |  | -- |
|  |  |  |  |
| Region |  |  |  |
| Northeast | -- |  | Ref. |
| North Central | -- |  | 0.41 (0.40, 0.42) |
| South | -- |  | 0.78 (0.77, 0.80) |
| West | -- |  | 0.55 (0.54, 0.56) |
| Unknown | -- |  | 0.68 (0.60, 0.79) |
|  |  |  |  |
| Clinical Characteristics |  |  |  |
| Alcohol use disorder | 1.29 (1.19, 1.39) |  | 1.45 (1.26, 1.66) |
| Opioid use disorder | 3.38 (3.24, 3.54) |  | 2.40 (2.15, 2.68) |
| Severe mental illness | 1.33 (1.30, 1.37) |  | 1.17 (1.13, 1.21) |
| Obesity | 1.06 (1.04, 1.08) |  | 1.14 (1.12, 1.16) |
| HIV infection | 5.07 (4.31, 5.97) |  | 2.61 (2.14, 3.18) |
| Pre-eclampsia | 1.11 (1.06, 1.17) |  | 1.08 (1.04, 1.13) |
| High-risk pregnancy | 1.50 (1.48, 1.53) |  | 1.25 (1.24, 1.27) |
| Gestational diabetes | 0.94 (0.91, 0.97) |  | 1.01 (0.99, 1.03) |
| Preterm labor | 1.01 (0.99, 1.04) |  | 1.00 (0.98, 1.02) |
| Multiple gestation | 0.87 (0.82, 0.92) |  | 1.12 (1.08, 1.16) |
|  |  |  |  |
| Obstetric panel | -- |  | -- |
| Tdap vaccination | 0.82 (0.80, 0.84) |  | 0.94 (0.93, 0.96) |
| Influenza vaccination | 0.9 (0.88, 0.92) |  | 0.94 (0.93, 0.96) |

*Note.* OR = odds ratio, CI = confidence interval.

^a^ Models included all listed variables.

| Table S6. Adjusted marginal probabilities of hepatitis C testing during pregnancy | | | |
| --- | --- | --- | --- |
|  | **Medicaid** |  | **Commercial** |
|  | % (95% CI) |  | % (95% CI) |
| Age Group |  |  |  |
| 15-18 | 15.9 (15.6, 16.2) |  | 19.3 (18.4, 20.2) |
| 19-29 | 14.4 (14.3, 14.5) |  | 17.8 (17.6, 17.9) |
| 30-39 | 12.4 (12.3, 12.5) |  | 16.9 (16.8, 17.0) |
| 40-44 | 9.9 (9.5, 10.2) |  | 16.5 (16.2, 16.8) |
|  |  |  |  |
| Race/Ethnicity |  |  |  |
| Black | 13.2 (13.1, 13.3) |  | -- |
| Hispanic | 7.2 (7.0, 7.4) |  | -- |
| White | 15.5 (15.4, 15.6) |  | -- |
| Other | 13.9 (13.7, 14.1) |  | -- |
| Unknown | 13.8 (13.3, 14.2) |  | -- |
|  |  |  |  |
| Region |  |  |  |
| Northeast | -- |  | 24.7 (24.5, 24.9) |
| North Central | -- |  | 11.4 (11.3, 11.5) |
| South | -- |  | 19.7 (19.6, 19.8) |
| West | -- |  | 13.6 (13.4, 13.7) |
| Unknown | -- |  | 19.3 (17.9, 20.7) |
|  |  |  |  |
| Clinical Characteristics |  |  |  |
| Alcohol use disorder | 18.7 (17.9, 19.4) |  | 23.1 (24.1, 25.0) |
| Opioid use disorder | 40.8 (40.2, 41.4) |  | 34.5 (32.8, 36.2) |
| Severe mental illness | 18.6 (18.4, 18.9) |  | 20.4 (20.1, 20.8) |
| Obesity | 15.5 (15.3, 15.7) |  | 19.7 (19.5, 19.9) |
| HIV infection | 47.0 (44.6, 49.5) |  | 32.1 (29.4, 35.0) |
| Preeclampsia | 14.5 (14.1, 14.9) |  | 18.7 (18.2, 19.1) |
| High-risk pregnancy | 18.8 (18.6, 18.9) |  | 19.9 (19.8, 20.0) |
| Gestational diabetes | 13.7 (13.5, 13.9) |  | 17.7 (17.5, 17.9) |
| Preterm labor | 14.6 (14.4, 14.8) |  | 17.8 (17.6, 18.1) |
| Multiple gestation | 12.7 (12.3, 13.1) |  | 18.9 (18.5, 19.4) |
|  |  |  |  |
| Obstetric panel | 19.9 (19.8, 20.0) |  | 22.3 (22.2, 22.4) |
| Tdap vaccination | 13.7 (13.5, 13.8) |  | 17.4 (17.3, 17.5) |
| Influenza vaccination | 14.5 (14.3, 14.7) |  | 18.3 (18.2, 18.4) |
